# Supplementary figures and images for: The impact of sprint interval training versus moderate intensity continuous training on blood pressure and cardiorespiratory health in adults: a systematic review and meta-analysis
Source: PeerJ. 2024 Mar 14;12:e17064. doi: 10.7717/peerj.17064 (PMC10944631; doi:10.7717/peerj.17064)

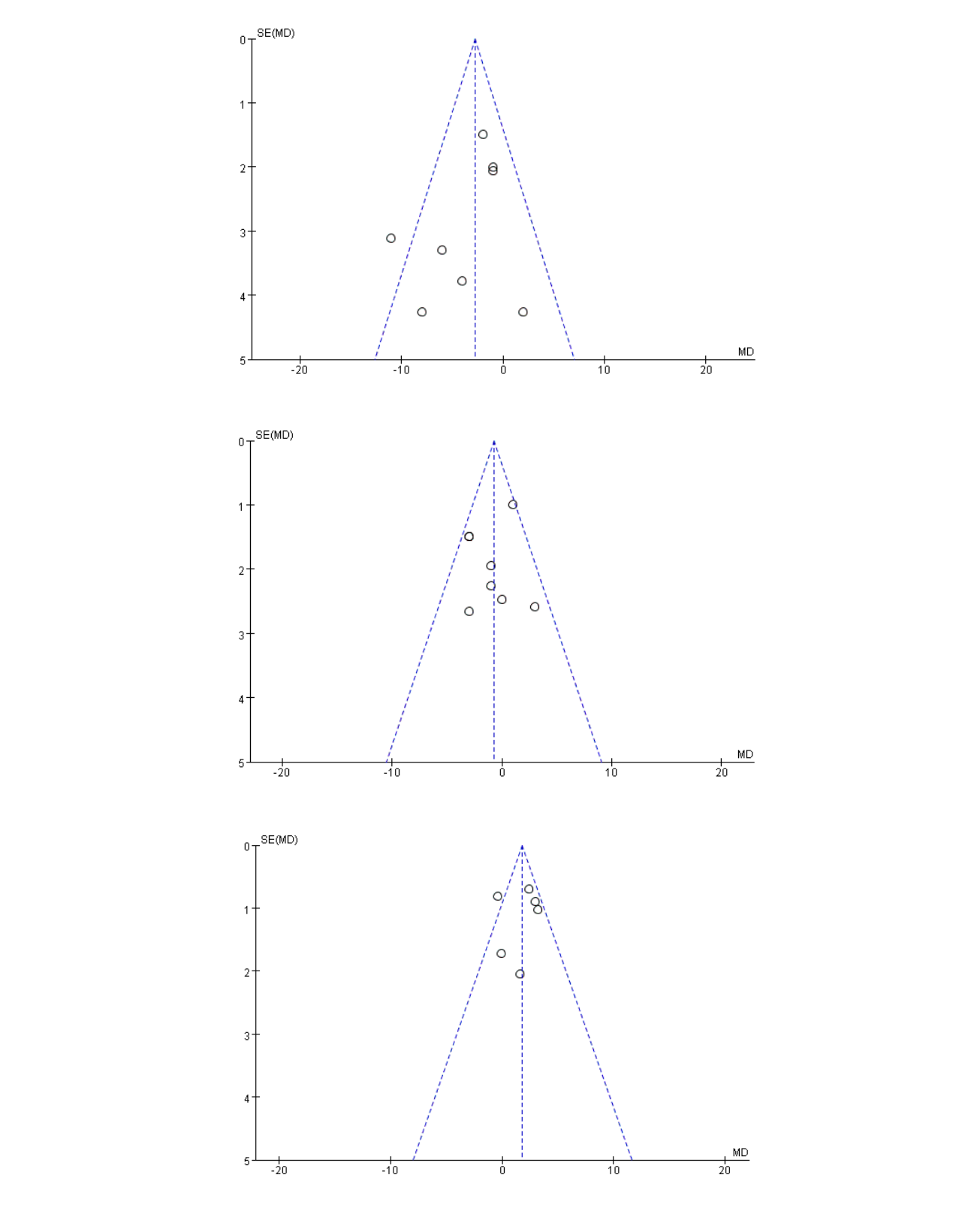

Supplement: Figure S1 — MD mean difference, SE standard error. [file peerj-12-17064-s002.png]

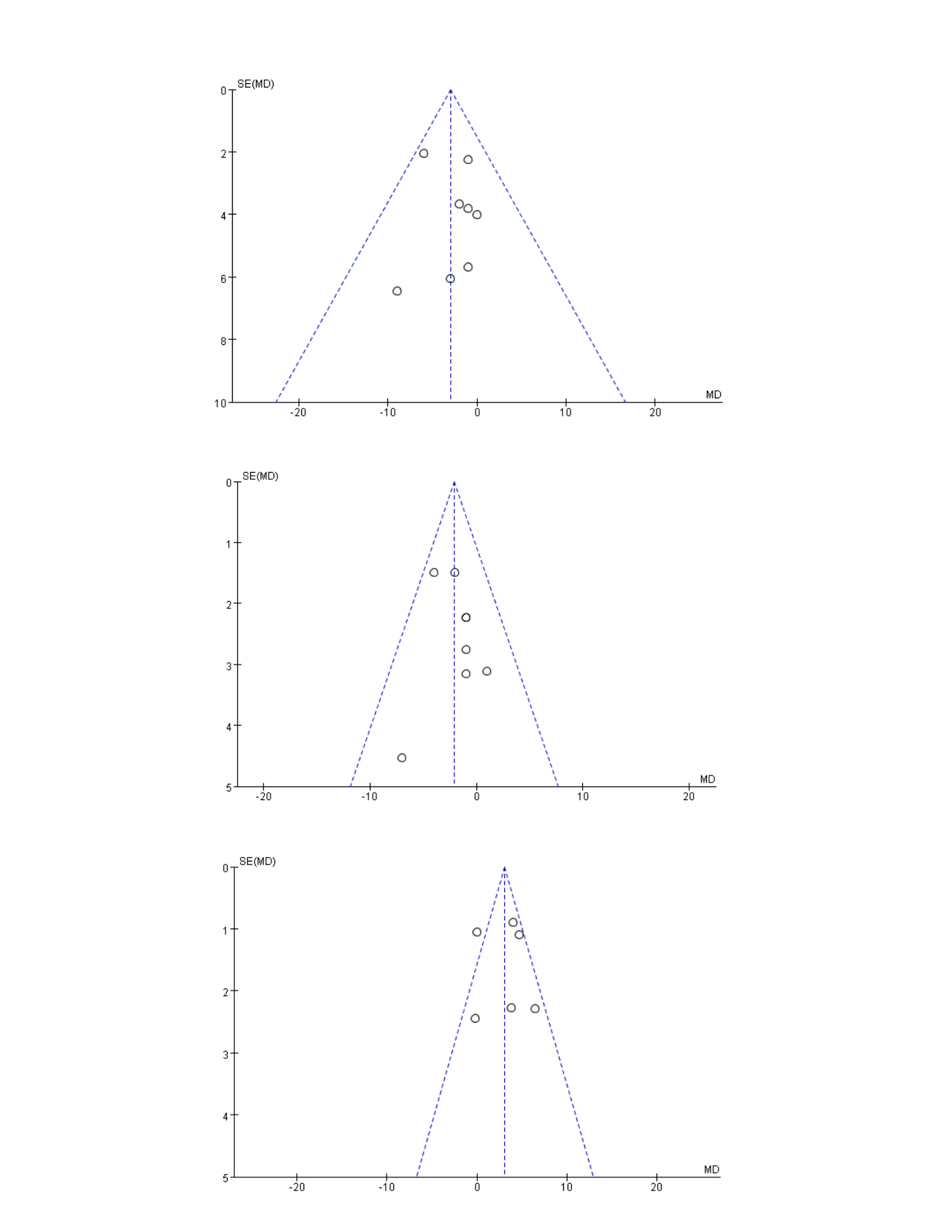

Supplement: Figure S2 — MD mean difference, SE standard error. [file peerj-12-17064-s003.png]

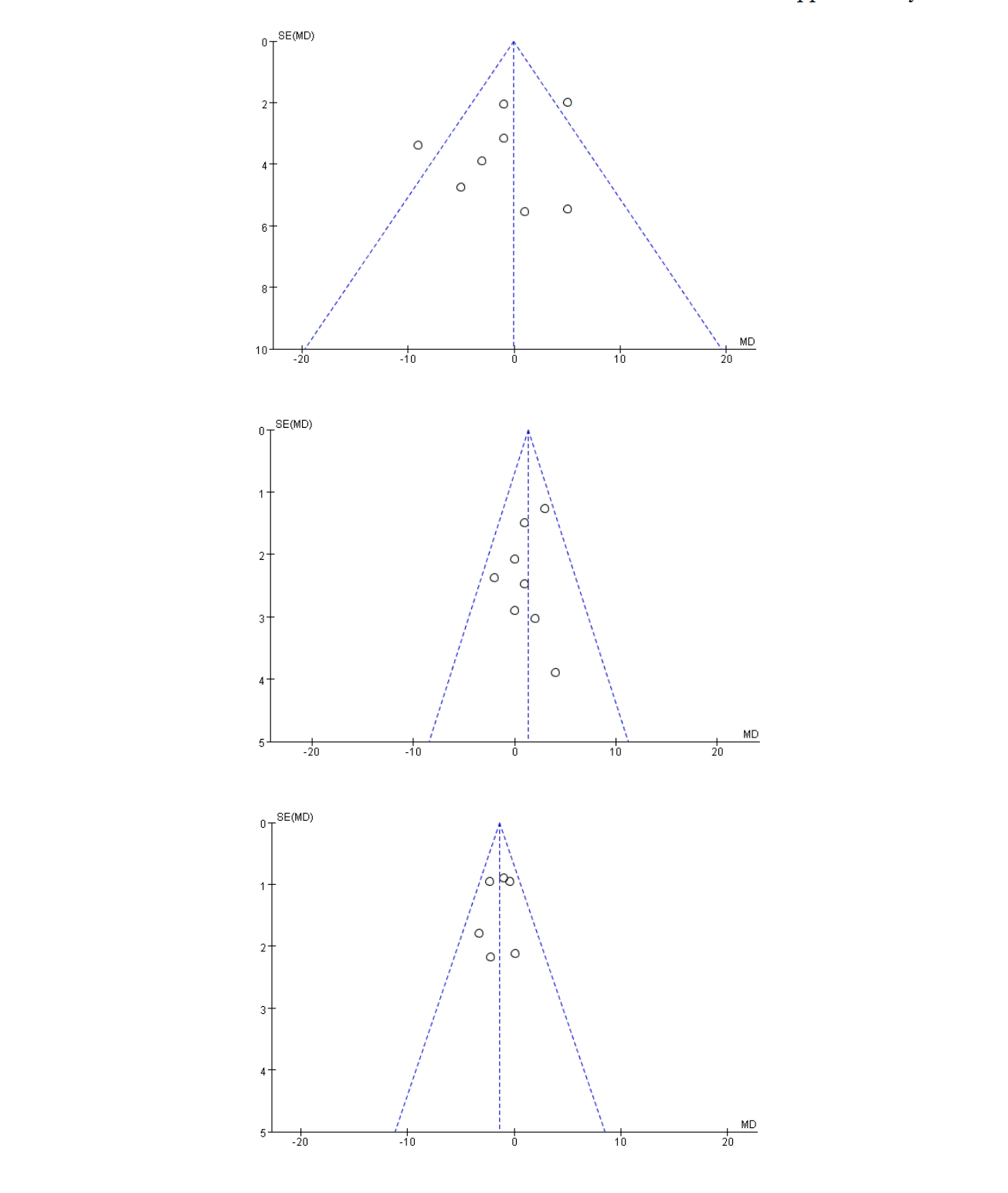

Supplement: Figure S3 — MD mean difference, SE standard error. [file peerj-12-17064-s004.png]
