# Supplementary material for: The impact of sprint interval training versus moderate intensity continuous training on blood pressure and cardiorespiratory health in adults: a systematic review and meta-analysis
Source: PeerJ. 2024 Mar 14;12:e17064. doi: 10.7717/peerj.17064 (PMC10944631; doi:10.7717/peerj.17064)
Supplement: Supplemental Information 5 [file peerj-12-17064-s005.docx]

**Systematic Review and Meta-Analysis Rationale**

More than 1 billion people worldwide have pre-hypertension or high blood pressure, which is a significant risk factor for cardiovascular disease, stroke and other health problems. Exercise can prevent and treat high blood pressure, but most modern people lack sufficient time, which reduces adherence to exercise. Sprint Interval Training (SIT) is a highly effective subcategory of interval training that consists of short bursts of high-intensity exercise. It has been shown to be efficacious in enhancing physical fitness and function. Such interventions may offer an efficient and diverse exercise prescription option. However, no previous relevant studies have explored this approach.

Numerous studies have reported comparable or greater improvements in various physical health indicators with SIT compared to traditional exercise modalities such as Moderate intensity continuous exercise(MICT). However, the effects of SIT and MICT on blood pressure are unknown. Therefore, the aim of this systematic review was to collect and evaluate all relevant studies that examined blood pressure and cardiorespiratory fitness responses to SIT in normotensive and hypertensive populations and to compare them with MICT protocols.
